# Breakfast and Other Meal Consumption in Adolescents from Southern Poland

**DOI:** 10.3390/ijerph13050453

**Published:** 2016-04-28

**Authors:** Agnieszka Ostachowska-Gasior, Monika Piwowar, Jacek Kwiatkowski, Janusz Kasperczyk, Agata Skop-Lewandowska

**Affiliations:** 1 Department of Hygiene and Dietetics, Medical College, Jagiellonian University, 7 Kopernika St., Krakow 31-034, Poland; jackwi@poczta.onet.pl (J.K.); agata.skop-lewandowska@uj.edu.pl (A.S.-L.); 2 Department of Bioinformatics and Telemedicine, Medical College, Jagiellonian University, 16 Lazarza St., Krakow 31-034, Poland; mpiwowar@cm-uj.krakow.pl; 3 School of Medicine with the Division of Dentistry in Zabrze, Medical University of Silesia, 19 Jordana St., Zabrze 41-808, Poland; jkas@mp.pl

**Keywords:** adolescents, eating behavior, breakfast skipping, meal consumption habits

## Abstract

The aim of the study was to evaluate the frequency of breakfast and other meal consumption by adolescents and to assess the relationship between the first and the last meal consumption and sex, body mass index (BMI), and middle school and high school students’ education level. The study was conducted in 2013–2014 among 3009 students (1658 girls and 1351 boys) from middle s and high schools in Krakow and Silesia (Poland). The data was obtained from questionnaires that were analyzed with a logistic regression model for measurable and dichotomous variables. Breakfast consumers were seen to eat other meals (second breakfast, lunch, dessert, supper) significantly more often than breakfast skippers. The main meal consumption habits depend on sex and change as adolescents age. Being a girl and a high school student predisposed participants to skip breakfast and supper more often. The BMI of breakfast consumers does not differ significantly from the BMI of breakfast skippers, so BMI might thus not be a sufficient marker of breakfast consumption regularity and dietary habits in an adolescent group. The importance of regularly eaten meals, especially breakfast, together with adequate daily dietary energy intake are beneficial for physical and psychological development and cannot be overestimated in nutritional education and it is necessary to promote healthy eating behavior for well-being in later adult life.

## 1. Introduction

Adolescence is a particularly unique period of life because it is a time of intense physical, psychosocial and cognitive development. The peak of growth is generally between 11 and 15 years for girls and 13 and 16 years for boys. During this period adolescents gain up to 50% of their adult weight, more than 20% of their adult height, and 50% of their adult skeletal mass [1]. Therefore, a balanced diet and eating meals regularly are both extremely important elements during this period of life [2,3]. Adolescence is also a time of greater autonomy, changing lifestyle and dietary habits. Those changes affect both nutrient needs and intake [4,5]. Some dietary patterns appear to be quite common among adolescents: frequent snacking (usually energy-dense foods); habitual meal skipping (particularly breakfast); late night dinner eating or irregular meal consumption; wide consumption of fast and highly processed foods and unconventional dietary practices [6,7]. Regardless of family income, adolescents of both sexes are at the risk of both dietary excesses (total fat, saturated fatty acids, cholesterol, sodium, table sugar) and deficiencies (fruit, vegetables, iron and calcium-rich foods) [8,9]. In Poland the majority of rural schoolchildren and adolescents aged 9–13 have poor dietary habits, including breakfast skipping, high consumption of sweets, and low consumption of fruit and vegetables [10,11]. There is a strong positive association between irregular breakfast eating and the risk of excess weight gain as the first step to obesity and metabolic disorders, diabetes, cardiovascular diseases [12,13,14,15,16,17,18]. In this aspect, the maintenance of balance between energy intake/output and adequate food daily intake is significant and breakfast has been considered to be an important factor for energy intake regulation [19]. Reduced breakfast energy intake is associated with higher total daily energy intake and when breakfast is skipped it can be difficult to compensate later during the day [20]. Studies have shown that breakfast is an important source of energy and nutrients after an overnight fast and can be helpful in achieving better concentration and better performance at school [21,22]. All environmental determinants which significantly lower their risk of excess weight gain are considered to be helpful for adolescent psychical and physical development [14,23,24]. Family and school play a significant role. It is confirmed that regular family meals positively impact the adolescent diet quality. Regular family meals during the transition from middle school to high school are associated with greater frequency of consuming breakfast and dinner meals and increased intakes of vegetables, dietary fiber, and several key nutrients, including calcium, magnesium, potassium, iron, and zinc [25]. School is the next place that influences the adolescent lifestyle, nutritional habits and meal patterns. School nutrition programs were launched in order to improve quality of school meals and to educate students about the impact of food on their health [26,27,28].

The main purpose of the study presented here was to evaluate the frequency of regular consumption of meals (breakfast, second breakfast, lunch, dessert and supper) by adolescents attending middle schools and high schools in Poland. A secondary purpose has been to evaluate the relationship between the first and the last meal (breakfast and supper) consumption and sex, BMI and level of education.

## 2. Materials and Methods

### 2.1. Survey Design and Sample

The examination included 3009 students from two university towns in Southern Poland: Krakow and Zabrze Silesia. The group comprised 1658 girls and 1351 boys aged 13–17 (mean age 15.95 ± 1.58) and with BMI 20.9 ± 3.2 kg/m^2^. A survey was carried out between September 2013 and June 2014 (Table 1) using an anonymous standardized self-completed questionnaire concerning dietary habits. All students were explained the purpose of the survey and the privacy protection policy of personal and enrollment data were ensured. All subjects gave their informed consent for inclusion before they participated in the study, which was conducted in accordance with the Declaration of Helsinki, and The Committee for Human Research of Jagiellonian University, Medical College in Krakow approved the protocol. In accordance with Polish legal regulations this kind of study is not considered a medical experiment and Ethical Committee agreement was not obligatory for medical doctors and dentists (“Dziennik Ustaw” 2011 with modifications; No. 277, line 1634). Participants completed the questionnaire in the classroom, and were provided with a verbal description of the study, and given the chance to ask questions prior to participation. The definition of each meal together with the indication of time during a day when it is usually consumed was presented in the questionnaire. The main meals (breakfast, lunch, supper) were defined as meals being the source of protein, fats, carbohydrates. Breakfast (more than a glass of milk or juice), as a meal that is eaten within one hour after getting up. Second breakfast, as a small meal before midday. Lunch, in Poland, defined as a hot dish consumed between 1 p.m. and 5 p.m. Dessert as a sweet meal or a fruit and supper as an evening meal providing about 20% of daily energy intake. Participants answered questions about the quantity of meals they usually consume. They were also asked to describe the composition of each meal, but the information about the serving size was not obligatory. Adolescents who did not consume breakfast at all or less than three times a week were categorized as breakfast skippers, and the rest of participants were classified as breakfast consumers. The same rules of qualification have been taken into consideration during the estimation of other meals consumption. Height and weight measurements have been taken by trained research staff using standardized protocols. Participants had an empty bladder, did not eat or drink anything three hours before measurements, and were asked to remove shoes and heavy garments before the measurement. Body weight has been measured to the nearest 0.1 kilogram using a digital scale (Model 882; Seca Corporation, Hamburg, Germany). Body height has been measured to the nearest 0.1 centimeter using a free-standing portable stadiometer (Model 214; Seca Corporation). From body weight and body height Body Mass Index (BMI) was calculated using a standard formula: weight (kg)/height (m)^2^. Acquired data was compared against the baseline values for a population of Polish adolescents [29]. Percentiles specific to age and sex classify children as underweight (<5th percentile), healthy weight (5th–85th percentile), overweight (85th to <95th) and obesity (≥95th percentile). Examined adolescents with BMI < 5th percentile and ≥ 85th percentile were classified as out of the norm.

### 2.2. Statistical Analysis

Differences between categorical variables have been tested by chi-square test (Table 2). Two models of multiple logistic regression analysis have been calculated:

#### 2.2.1. Model 1

Dependent variable: breakfast consumption.

Independent variables: sex, BMI and age range (expressed as level of education), second breakfast, lunch, dessert and supper consumption.

#### 2.2.2. Model 2

Dependent variable: supper consumption.

Independent variables: sex, BMI and age range (expressed as level of education), breakfast, second breakfast, lunch, dessert consumption.

Crude odds ratios (OR) have been calculated to evaluate the risk of independent variables and associated 95% confidence intervals. Multivariate logistic regression models have been used to adjust for the possible confounding influences among the independent variables on the dependent in model. Variables with significant independent predictive values (*p* < 0.05) were identified. A probability (*p*) value of <0.05 was regarded as statistically significant, *p*-value > 0.05 has been assigned as not significant (NS). Statistical analysis has been performed with R software—2.15.3 version [R Core Team (2015), R: A language and environment for statistical computing. R Foundation for Statistical Computing, Vienna, Austria. URL https://www.R-project.org/], GLM procedure.

## 3. Results

The descriptive characteristics of the participants are presented in Table 1.

### 3.1. BMI of Participants

The range of BMI in the examined adolescents is presented in Table 2. BMI out of the norm was observed for 18.7% of all examined students and 70.1% of them were overweight. Overweight was more often seen among boys. Both underweight and overweight adolescents were more often seen in the older age group (high schools students).

### 3.2. Sex and Age Differences in Meals Consumption

While the analysis was performed to evaluate the frequency of consumption or skipping particular meals with respect to age and gender, it was shown that breakfast, lunch and supper are regularly eaten only by some of the examined adolescents.

Breakfast was statistically significantly consumed more often by boys than by girls, and it was more typical of high school students. There were no significant differences in breakfast consumption between girls and boys from middle schools. Lunch has been the meal eaten most often, regardless of sex. Nevertheless, the percentage of adolescents consuming lunch decreased significantly in high school in proportion to age, a fact that was particularly noticeable among girls. In middle schools there were no differences between girls’ and boys’ lunch consumption. Supper was consumed significantly more often by boys, both from middle schools and high schools, with the differences between girls and boys becoming more visible in the older age group. In high school supper was skipped by 12.8% of girls and by 4.3% of boys. The proportion of girls who were seen to skip supper was higher, but the differences between younger and older girls were statistically non-significant. Second breakfast and dessert are the most frequent skipped meals. Second breakfast consumption was similar for all participants, regardless of their sex, but this meal has been omitted more frequently by students from middle schools. Nearly 40% of all questioned persons did not consume dessert. Dessert was skipped more often by girls, especially by high school girls In case of boys the tendency was reversed (Table 3).

### 3.3. Breakfast Consumption

Students who eat breakfasts regularly, were seen to eat other meals as well, regardless of education level and sex. Among breakfast consumers there were both girls and boys, although being a girl predisposed to skip breakfast (OR = 0.69). The level of education has been a differentiating variable—older teenagers consumed breakfasts more rarely than younger students. Students attending middle school ate breakfast nearly twice as often in comparison to high school students (OR = 1.66). Breakfast consumers were seen to eat supper three times more often (OR = 3.0), lunch almost three times more often (OR = 2.9) and second breakfast and dessert about one and a half time more often than breakfast skippers. There were no differences in BMI range between breakfast consumers and breakfast skippers (Table 4).

### 3.4. Supper Consumption

Supper was skipped significantly more often by girls (OR = 0.35) than boys. BMI values were not influenced the supper consumption (OR = 0.97). Supper was eaten nearly three times more often (OR = 2.91) by breakfast consumers than breakfast skippers, and was consumed twice as often by persons who has eaten a second breakfast and dessert. Eating lunch had the biggest influence on supper consumption. Those persons ate supper nearly five times more often (OR = 4.86) in comparison to persons who were seen to skip lunch (Table 5).

## 4. Discussion

In Poland the National Nutrition Institute in Warsaw and the Polish Ministry of Health have created Food-Based Dietary Guidelines (FBDGs), which are disseminated in the form of 10 guidelines on healthy diet, healthy nutrition pyramid and general directives (http://www.izz.waw.pl/en/). According to the guidelines five daily meals are recommended and three main meals (breakfast, lunch, supper) are regarded as particularly important. The results of the presented study indicate that lunch is the meal eaten most often by adolescents, regardless of their sex, whilst the rest of meals are skipped more often by girls than boys. The habit of main meals being omitted is different among girls and boys and furthermore changes as they are getting older—adolescents from high schools eat main meals less often than adolescents from middle schools. Compared to younger children, worse food habits in adolescents could be explained by a higher degree of autonomy at this age and more freedom of decision making. The findings regarding age and gender variations are consistent with results from other countries [22,30,31,32]. The Health Behavior in School-aged Children (HBSC) results inform that about 30% of 11–15 year old Polish adolescents do not eat breakfast every morning on school days and 20%–30% do not have any meal during school hours [33]. Breakfast is not consumed regularly by 12.5% of girls and 9.3% of examined boys. Among this group there were persons with BMI recommended for that age and sex and with BMI out of the norm, so normal BMI is not a sufficient marker of breakfast consumption habits Underweight participants (3.8% of all examined) have also reported not consuming breakfast and other meals. It is possible that within this group persons at risk of developing eating disorders might be found. Overweight has been recognized in 14.9% of the examined students and mostly among boys. The 2009/2010 report of the Polish Institute of Mother and Child has stated that overweight is more frequent among boys, and the results of this paper seem to confirm that tendency [7]. Numerous studies have concluded that eating breakfast may reduce the risk of excess weight gain [34,35]. Several authors have demonstrated a relationship between skipping breakfast and higher BMI among different populations of children [36,37,38]. This study does not show such a strong correlation between overweight and breakfast skipping. BMI of breakfast consumers was only slightly lower than that of breakfast skippers’ (OR = 0.96; *p* < 0.042). The reason of overweight may be an excess intake of calories, that starts with breakfast. Normal BMI among breakfast skippers might indicate that their energy requirements are being satisfied by meals other than breakfast. The study was designed to evaluate the quantity of consumed meals, but the energy intake from diet has not been taken under consideration and should be taken into account in any further research on this topic. Breakfast skipping by some students with healthy BMI might be considered as the first sign of poor dietary habit development and the consequences of not eating breakfast might become visible in their adult life, when changes leading to lower metabolic rate, lower level of physical activity, and endocrine disruption occur. Childhood dietary habits are hard to break in future adult life and this is why it is essential to also intensify nutritional education among adolescents with normal BMI. It is important to keep in mind that BMI is only a proportion of total body weight to height, and among persons with normal BMI there can be those with unhealthy proportions between lean body mass and fat mass. Therefore methods evaluating body composition should be considered as a better measure than BMI in assessing nutritional status and maybe in assessing dietary habits. Special nutritional programs are created in Poland and in other countries to fight overweight and underweight among students and protect them from dangerous consequences of unhealthy dietary habits for their physical and mental development.

In the United States, School Breakfast Programs (SBP) have been created and schools have received subsidies for each breakfast they serve for students [27]. Low income students may eat free or reduced-cost breakfasts in this way. School participation varies by state and region. Some participating schools offer free breakfast to all students, others only to qualifying students. Breakfast can be served in the cafeteria before school starts or in classrooms as the school day begins [26]. In the United States nearly 90,000 schools participated in the SBP in the 2011–2012 school year and approximately 12.5 million children got free meals each day. About half of the low income children participated in the SBP [39]. There is a strong evidence that having access to the School Breakfast Program (SBP) cognition, attendance, and scholastic achievement, especially in nutrition of deficient or malnourished children may be improved [40,41]. High-quality universal breakfast programs that allow students to eat breakfast in the classroom are especially needed for youth who are not likely to get good food for the rest of a day [28]. There is a great diversity of programs and structures for healthy eating in schools in Europe but a national comprehensive policy regarding the prevention of obesity in schools does not yet exist in every European country [21,42,43]. To help deal with this issue the Healthy Eating and Physical Activity in Schools (HELPS) project has been developed to support countries in Europe in promoting healthy eating and physical activity at schools in a positive and sustainable way. This project is linked to the Schools for Health in Europe network [44].

In Poland, schools are obliged to carry out health education, including nutritional education [45,46]. In the new core curriculum, implemented in 2008, there are many topics related to healthy eating. The organization of school meals for all students, as well as the participation of schools in obesity prevention programs were not satisfactory, but now activities to prevent overweight are specified in the 2007–2015 National Health Program [47]. Information obtained from 520 randomly selected primary, lower-secondary and cluster schools from all the voivodeships of Poland showed that in some ongoing national programs like “A Glass of Milk” the participation rate was 74% (only 25% of the lower secondary schools) and in “Fruit at School” 38% (6% of lower secondary schools) respectively. The “Keep Fit” educational program was implemented in 28% of primary schools and in 72% of lower secondary schools [45]. Nevertheless the majority of schools in Poland still treat students’ need to eat a meal at school as a minor problem and are not involved in any activities focusing on prevention of obesity. The undertaken activities are incoherent and there is a need to create a healthy nutrition policy on the national, regional, local and school level.

## 5. Conclusions

The findings have confirmed the importance of proper nutritional education among adolescents and indicate that students who consumed breakfast regularly are also likely to eat other meals. This fact might lead to conclusion that breakfast consumption encourages consumption of more meals during a day, but as obesity has become a global issue it is necessary to pay more attention to energy and nutrient intake. The habits of main meals consumption differ among girls and boys and changes as they get older—adolescents from high schools eat main meals less often than middle school students. BMI of breakfast consumers and non-consumers did not differ significantly in the examined adolescents—this might suggest that BMI is an insufficient marker of regularity of breakfast consumption and the dietary habits of adolescents. It is likely that the consequences of not consuming breakfast will be recognized in the nutritional status during future adult life, when breaking dietary habits is more difficult. To verify that thesis future research will be undertaken.

## Figures and Tables

**Table 1:** Age and anthropometric data for adolescents enrolled in the study (*n* = 3009).

Kind of School		Sex				Σ	Measurable Variable (mean ± SD)
		Female		Male			
		*n*	Measurable Variable (mean ± SD)	*n*	Measurable Variable (mean ± SD)		
School	High school	1160	Weight: 57.1 ± 8.1 (kg)	1004	Weight: 71.4 ± 12.1 (kg)	2164	Weight: 60.8 ± 12.7 (kg) Height: 169.9 ± 9.5 (cm) BMI: 20.9 ± 3.2 (kg/m^2^) Age: 16.8 ± 0.80 (years)
			Height: 166.5 ± 6.0 (cm)		Height: 179.1 ± 6.8 (cm)		
			BMI: 20.6 ± 2.7 (kg/m^2^)		BMI: 22.2 ± 3.3 (kg/m^2^)		
			Age: 16.8 ± 0.8 (years)		Age: 16.8 ± 0.80 (years)		
	Middle school	498	Weight: 50.9 ± 8.4 (kg)	347	Weight: 56.6 ± 12.0 (kg)	845	
			Height: 161 ± 7.0 (cm)		Height: 166.5 ± 7.9 (cm)		
			BMI: 19.3 ± 2.6 (kg/m^2^)		BMI: 20.3 ± 3.5 (kg/m^2^)		
			Age: 13.8 ± 1.0 (years)		Age: 13.9 ± 1.0 (years)		
Σ n		1658	-	1351	-	3009	

**Table 2:** BMI range in examined adolescents from middle and high schools.

Adolescents			BMI	
**Sex and Level of Education**		**Underweight *n***	**Normal *n***	**Overweight *n***
Female	Middle school	11	458	29
	High school	83	924	153
Male	Middle school	7	287	53
	High school	14	778	212
**All Examined Σ n (%)**		**115 (3.8%)**	**2447 (81.3%)**	**447 (14.9%)**
All examined female Σ n		94	1382	182
All examined male Σ n		21	1065	265

Underweight—BMI < 5th percentile; Normal—BMI 5th–85th percentile; Overweight—BMI ≥ 85th percentile.

**Table 3:** The proportion between consumers and non-consumers of particular meals with regard to level of education.

Meals	All Examined Adolescents		Middle School		High School		Significance *p*-Value
	F (■)	M (■)	F (♦)	M (♦)	F (●)	M (●)	
Breakfast	Y = 87.5 N = 12.5	Y = 90.7 N = 9.3	Y = 91.4 N = 8.6	Y = 93.9 N = 6.01	Y = 85.9 N = 14.1	Y = 89.6 N = 10.4	F(**■**)/M(**■**) 0.006 F(●)/M(●) 0.009 F(♦)/M(♦) NS F(●)/F(♦) 0.002 M(●)/M(♦) 0.022
Second Breakfast	Y = 77.5 N = 22.5	Y = 75.8 N = 24.2	Y = 73.5 N = 26.5	Y = 68.6 N = 31.4	Y = 79.2 N = 20.8	Y = 78.3 N = 21.7	F(**■**)/M(**■**) NS F(●)/M(●) NS F(♦)/M(♦) NS F(●)/F(♦) 0.010 M(●)/M(♦) 0.0003
Lunch	Y = 94.1 N = 5.9	Y = 97.9 N = 2.1	Y = 96.2 N = 3.8	Y = 98.0 N = 2.0	Y = 93.3 N = 6.7	Y = 97.9 N = 2.1	F(**■**)/M(**■**) 7.2 × 10^−7^ F(●)/M(●) 9.2 × 10^−7^ F(♦)/M(♦) NS F(●)/F(♦) 0.012 M(●)/M(♦) 0.0003
Dessert	Y = 60.9 N = 39.1	Y = 64.5 N = 35.5	Y = 63.1 N = 36.9	Y = 58.8 N = 41.2	Y = 59.9 N = 40.1	Y = 66.4 N = 33.6	F(**■**)/M(**■**) 0.046 F(●)/M(●) 0.002 F(♦)/M(♦) NS F(●)/F(♦) 0.008 M(●)/M(♦) 0.012
Supper	Y = 88.5 N = 11.5	Y = 95.7 N = 4.3	Y = 91.8 N = 8.2	Y = 95.7 N = 4.3	Y = 87.2 N = 12.8	Y = 95.7 N = 4.3	F(**■**)/M(**■**) 1.8 × 10^−12^ F(●)/M(●) 4.8 × 10^−12^ F(♦)/M(♦) 0.03 F(●)/F(♦) NS M(●)/M(♦) NS

F—Female, M—Male; Y—% of consumers; N—% of non-consumers; NS—Not significant.

**Table 4:** Adjusted odds ratios for breakfast consumption with regard to sex, BMI, age (expressed as level of education) and other meals consumption.

Variables	OR	2.5%	97.5%	*p* Value
Sex (Female)	0.69	0.51	0.83	0.0007
BMI	0.96	0.92	0.99	0.0421
School (Middle school)	1.66	1.25	2.24	0.0006
Second breakfast (Y)	1.59	1.23	2.04	0.0003
Lunch (Dinner) (Y)	2.88	1.87	4.35	7.7 × 10^−7^
Dessert (Y)	1.51	0.17	0.66	0.0007
Supper (Y)	3.01	2.17	4.13	1.7 × 10^−11^

Y—consumers.

**Table 5:** Adjusted odds ratios for supper consumption with regard to sex, BMI, age (expressed as level of education) and other meals consumption.

Variables	OR	2.5%	97.5%	*p* Value
Sex (Female)	0.35	0.25	0.48	3.6 × 10^−10^
BMI	0.97	0.92	1.01	NS
School (High school)	1.48	0.84	2.67	NS
Breakfast (Y)	2.91	2.09	4.00	1.1 × 10^−10^
Second breakfast (Y)	1.98	1.48	2.63	3.5 × 10^−6^
Lunch/Dinner (Y)	4.86	3.19	7.32	7.6 × 10^−14^
Dessert (Y)	2.05	1.54	2.72	5.8 × 10^−7^

Y—consumers.
